# Improving kidney transplant care through the application of continuous glucose monitoring - a narrative review

**DOI:** 10.3389/fneph.2025.1630597

**Published:** 2025-11-28

**Authors:** Jackson Tan, Muhammad Abdul Mabood Khalil, Parizad Avari, Lalantha Leelarathna

**Affiliations:** 1 Department of Renal Medicine, Raja Isteri Pengiran Anak Saleha Hospital, Bandar Seri Begawan, Brunei; 2 PAPRSAB Institute of Health Sciences, Universiti Brunei Darussalam, Bandar Seri Begawan, Brunei; 3 Department of Renal Medicine, King Fahad General Hospital, Jeddah, Saudi Arabia; 4 Department of Metabolism, Digestion and Reproduction, Imperial College, London, United Kingdom; 5 Imperial College Healthcare NHS Trust, London, United Kingdom

**Keywords:** continuous glucose monitoring, CGM, kidney transplant, kidney failure, diabetes mellitus, post-transplant diabetes mellitus, artificial intelligence

## Abstract

Continuous glucose monitoring (CGM) is used more frequently among patients with chronic kidney disease (CKD), including those undergoing haemodialysis and peritoneal dialysis. However, there is a lack of information and evidence regarding CGM use in kidney transplantation (KT). Dysglycaemia is commonly observed in the transplant setting; often complicated by impaired kidney function with fluctuating glomerular filtration rates and competing influences of diabetogenic immunosuppressants, perioperative surgical stress and transplant-related complications. This narrative review, the first of its kind, examines the utility, accuracy, efficacy and clinical outcomes of CGM in KT patients. It also addresses specific transplant-related issues that may necessitate future CGM usage and highlights knowledge gaps to inform future research directions.

## Background

Globally, diabetes mellitus (DM) is the most prevalent cause of chronic kidney disease (CKD) and end stage kidney disease (ESKD) (1). Correspondingly, approximately 20-40% of all patients with DM will be affected by Diabetic Kidney Disease (DKD) (2). The incidence and prevalence of DM-related ESKD are increasing annually, in parallel with trends of DM; likely through the effects of urbanization (3). Amongst kidney transplant (KT) patients, the incidence of post-transplant diabetes mellitus (PTDM) ranges from 15-30% (4). Control of DM in CKD patients is often challenging due to the complex interplay between insulin metabolism and aberrant renal gluconeogenesis through metabolic acidosis, uraemia and chronic inflammation (5). In KT recipients, glycaemic control is further complicated by additional factors such as the diabetogenic effects of immunosuppressive therapy, surgical stress, and an increased susceptibility to infections.

Traditionally, glycated haemoglobin (HbA1c) is often regarded as the yardstick measure for long-term glycaemic control, but is fraught with intricacies regarding applicability and accuracy within the CKD population due to altered red blood cell turnover and anaemia (4). Furthermore, HbA1c provides no information about the frequency and the burden of hypoglycaemia or glucose variability. The Dialysis Outcomes and Practice Pattern Study (DOPPS) reported a U-shaped relationship between HbA1c and all-cause mortality, with increased risk of mortalities by 38% and 21% in dialysis patients with HbA1c >9% and < 7%, respectively, compared to patients with HbA1c of 7-8% (6). The 2022 Kidney Disease Improving Global Outcome (KDIGO) guideline recommended an optimal HbA1c range between 6.5-8.0% for non-dialysis patients with DKD, with higher HbA1c targets for more advanced stages of CKD (7). For KT patients with diabetes, the recommended target HbA1c is 7.0-7.5% and to specifically avoid Hba1c of < 6.0%, especially in patients with advanced CKD stages of KT (8). However, HbA1c levels can be less reliable in KT recipients, especially in the early post-transplant period (typically first three months), due to immediate changes in red blood cell turnover and dynamic fluid shift post-transplant. While HbA1c remains a valuable tool for long-term glycaemic assessment, its interpretation in this specific context requires caution, and alternative methods like continuous glucose monitoring (CGM) might be considered.

Continuous Glucose Monitoring (CGM) has revolutionized the surveillance and management of DM over the past decade (9). The use of CGM in people with insulin-treated diabetes has been shown to improve HbA1c, increase time spent in the target glucose range of 3.9 to 10 mmol/L, reduce risk of hypoglycaemia, and improve patient-reported outcome measures (10, 11). A recent meta-analysis encompassing 26 randomized controlled trials demonstrated that the use of CGM was associated with significant improvements in HbA1c levels and reductions in glycaemic medication requirements among patients with type 2 diabetes mellitus (T2DM) (12). Nonetheless, several caveats persist, including uncertainties regarding CGM accuracy due to discrepancies between interstitial and plasma glucose measurements, the influence of extreme temperatures and altered tissue perfusion, as well as potential complications related to sensor insertion, such as local inflammation and infection. A key advantage of CGM in CKD patients is the better assessment of true glycaemia control beyond the accepted paradigm of HbA1c and fasting blood sugar (FBS). CGM -metrics like time in range (TIR), time below range (TBR), time above range (TAR), glucose variability (GV), coefficient variation (CV), and glucose management indicator (GMI) provide a better overall assessment of glycaemia, particularly in situations when glycaemic control is unstable and dynamic. The recent KDIGO clinical practice guideline has recommended CGM to guide self-management when HbA1c is discordant with measured readings or to help prevent hypoglycaemia through predictive trends (7).

Previous reviews have focused on CGM research in CKD patients, including haemodialysis (HD) and peritoneal dialysis (PD) patients (13–15). A recent consensus report on CGM in CKD identified three main goals of management: encourage greater use of CGM in CKD patients, evaluate usage in ESKD patients and allow equitable access (16). The report also acknowledged that there is limited literature on CGM usage in KT, with recommendations to determine the accuracy of CGM and its impact on PTDM (16). This is the first-ever review on CGM usage in KT patients. The objectives of this review are to evaluate the benefits of CGM in KT patients with diabetes, discuss unique transplant issues that render the utility of CGM and identify knowledge gaps that may guide future research directions. In addition, it also enables the evaluation of CGM evidence in selected CKD and non-KT populations and extrapolate its applicability to KT patients.

## Challenges of glycaemic control in advanced CKD and transplant patients

Management of glycaemic control in CKD patients can be challenging with a high risk of hypoglycaemia and hyperglycaemia. HbA1c assessment is often influenced by anaemia, use of erythropoiesis agents and chronic inflammation (15). Other surrogate glycaemic markers like glycated albumin and fructosamine are affected by uraemia and abnormal albumin metabolism (15). Assessment of glycaemic control in the dialysis population is also complicated by unpredictable daily fluid volume shift, dialysers’ removal of drug and glucose and exposure to glucose-containing dialysate (5).

Many pharmacological, biochemical and surgical factors can influence dysglycaemia in KT. Glycaemic assessment using HbA1c may not accurately reflect the true glycaemic burden during the early post-transplant period (typically the first three months) due to several confounding factors, including recent blood transfusions, increased endogenous erythropoietin production from the newly functioning graft, fluctuating glomerular filtration rates, and the use of high-dose corticosteroids and calcineurin inhibitors (CNI) (17). The correlation between actual glucose levels and HbA1c measurements generally improves beyond the first three months post-transplant, coinciding with stabilization of graft function and reduced variability in haematologic and glycaemic parameters.

In experimental studies with mice, the administration of tacrolimus is associated with immediate impaired oral glucose tolerance test response, which indicate a propensity for postprandial hyperglycaemia (17). Methylprednisolone and prednisolone have a peak transient glycaemic action of 4–6 hours after administration, through the development of insulin resistance (18). Treatment with CNI and SGLT-2 may also predispose patients to diabetic ketoacidosis (DKA), even in those without a prior diagnosis of DM (19). Mammalian target of rapamycin inhibitor (mTORi), like everolimus and sirolimus, reduces peripheral resistance to insulin and insulin transduction signals leading to long-term dysregulated glucose control (20). The combination of CNI and mTORi is known to have a potent synergetic effect in the development of PTDM, compared to monotherapy (21). Similarly, steroids with CNI are also observed to have enhanced diabetogenic effects on patients (22). **Table 1** summarises the effects of transplant medications on glycaemic control.

**Table 1:** Type of transplant drugs uses and effects on glycaemia.

Drug	Timing of drug introduction	Duration of use	Mechanism of action in transplant	Effects on glycaemic pathway
Methylprednisolone	Intraoperative, before release of arterial clamps and treatment for acute cellular rejection	Few doses (depending on indications)	Suppress complement binding, enhance IL-10 expression, reduce IL-2, IL-6, and interferon γ expression in T-lymphocytes	Increase gluconeogenesis in liver, reduce insulin secretion in beta cells and reduce peripheral insulin sensitivity
Hydrocortisone	Intra- or postoperative, given together with anti-thymocyte globulin to prevent anaphylaxis	Few doses	Suppress complement binding, enhance IL-10 expression, reduce IL-2, IL-6, and interferon γ expression in T-lymphocytes	Increase gluconeogenesis in liver, reduce insulin secretion in beta cells and reduce peripheral insulin sensitivity
Prednisolone	Immediate postoperative	Long-term (usually reducing regimen with time)	Suppress complement binding, enhance IL-10 expression, reduce IL-2, IL-6, and interferon γ expression in T-lymphocytes	Increase gluconeogenesis in liver, reduce insulin secretion in beta cells and reduce peripheral insulin sensitivity. Has synergestic effects with CNI in causing PTDM
Tacrolimus	Immediate postoperative	Long-term (adjusted by drug level)	Reduce T-cell proliferation through binding to immunophilins which inhibit calcineurin	Reduce insulin release and synthesis. Additive effects with steroids and mTORi in causing PTDM
Ciclosporin	Immediate postoperative, long-term maintenance drug	Long-term (adjusted by drug level)	Reduce T-cell proliferation through binding to immunophilins which inhibit calcineurin	Reduce insulin release and synthesis. Additive effects with steroids and mTORi in causing PTDM
Everolimus	Usually introduced after a few weeks of transplantation	Long-term (adjusted by drug level)	Inhibit the activation of mTOR serine-threokine kinase which impairs proliferation of B and T cell lymphocytes	Reduce peripheral resistance to insulin, reduce insulin transduction signals and reduce beta cell proliferation. Has synergestic effects with CNI in causing PTDM.
Sirolimus	Usually introduced after a few weeks of transplantation	Long-term (adjusted by drug level)	Inhibit the activation of mTOR serine-threokine kinase which impairs proliferation of B and T cell lymphocytes	Reduce peripheral resistance to insulin, reduce insulin transduction signals and reduce beta cell proliferation. Has synergestic effects with CNI in causing PTDM.

**Figure 1:**
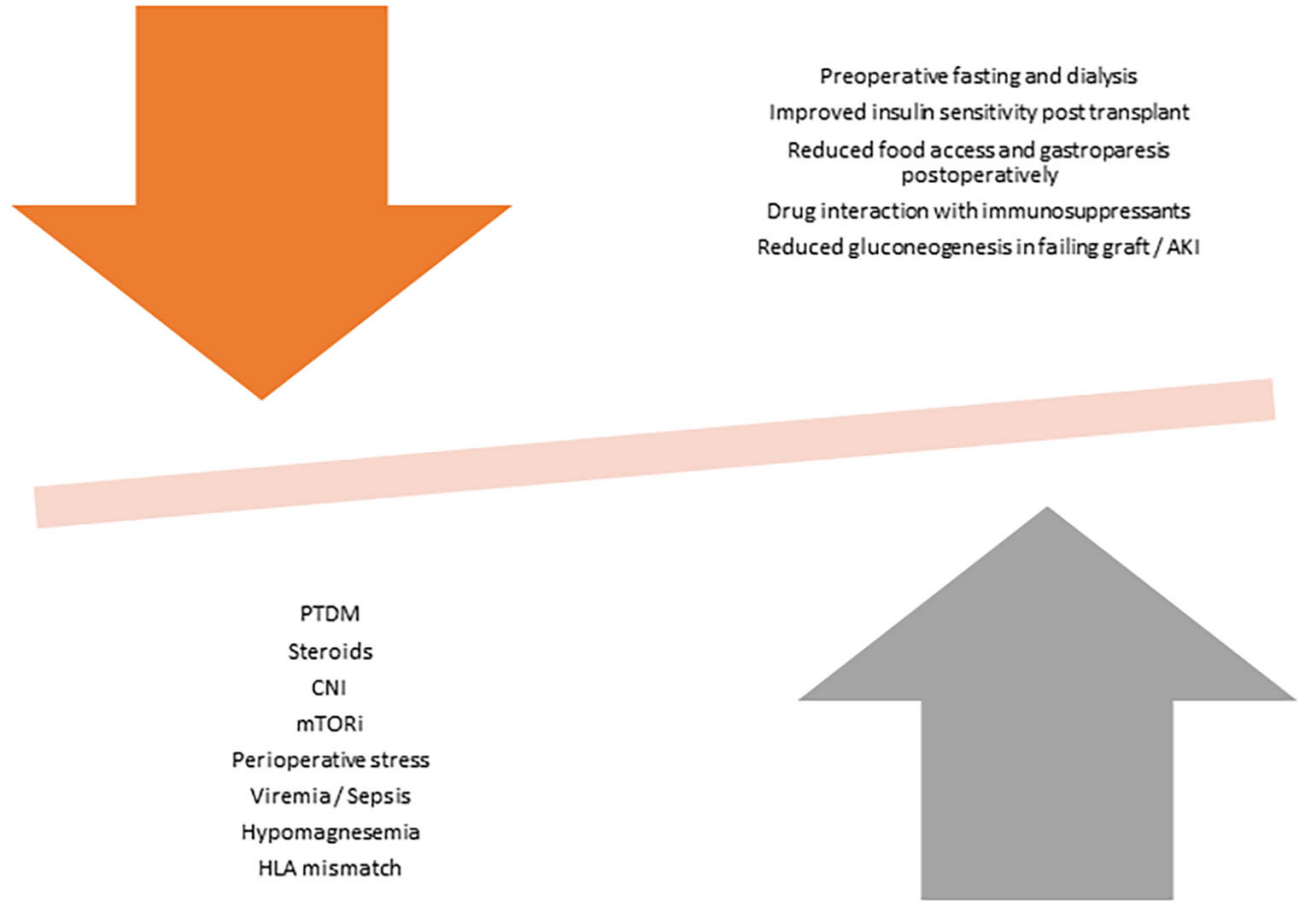
Transplant factors affecting hyperglycaemia and hypoglycaemia.

Non-pharmacological factors like surgical stress and pain can cause hyperglycaemia through secretion of cytokines and stress hormones that can affect insulin function (23). Improvement of kidney function in the post-operative phase improves insulin clearance, which may unmask preexisting hyperglycaemic tendencies (23). Acute or chronic hypomagnesemia, often seen in KT patients, has also been identified as an independent risk factor for PTDM through its role as an intracellular cofactor for glucose transport between membranes, glucose oxidation and insulin-mediated tyrosine kinase pathways (24). Asymptomatic Cytomegalovirus infection can cause impaired beta cell antiviral defence in the pancreas and increase risk of PTDM (25). Other transplant-related factors that increases risk of PTDM include male gender, HLA matching characteristics, hepatitis C infections and deceased donor kidney (16). **Figure 1** demonstrate the competing influences of KT-related factors on hyperglycaemia and hypoglycaemia.

## Use of CGM in advanced CKD (including transplant) and dialysis

The shortcomings of HbA1c as a glycaemic indicator in CKD patients provide opportunities for CGM to improve and complement existing measures. Amongst patients with ‘burnt-out’ diabetes in ESKD, CGM is reported to be superior to Hba1c and fructosamine in demonstrating undiagnosed hyperglycaemia and GV (26). The correlation between HbA1c and CGM metrics decreases with advanced stages of CKD (27), showing a good relationship between HbA1c and TAR and TIR, but not with TBR and hypoglycaemia (15). Glycaemic variability (GV), which has been shown to influence long-term adverse microvascular outcomes (28), has a poor correlation with HbA1c in both CKD (15) and non-CKD (29) populations. International consensus recommends that clinical targets for CGM data interpretation for high-risk patients include > 50% TIR (target range 3.9–10 mmol/l), < 1% TBR (< 3.9 mmol/l), and <10% TAR (>13.9 mmol/l) (30). However, these targets have not been validated in patients with CKD, and long-term hard outcomes data are still unavailable (16).

CGM devices measure glucose readings in interstitial fluid through an electrochemical reaction in the filament sensor located in subcutaneous tissues, which is determined by the dynamic gradient across the blood-interstitial barrier (31). Chronic hypoxia and anaemia in CKD patients interfere with the electrochemical sensing of the oxidase-peroxidase reaction and may explain the elevated Mean Absolute Relative Difference (MARD) values between CGM and capillary blood glucose readings (32, 33). Early sensors routinely had a %MARD greater than 20% but most commercially available sensors in people without advanced CKD or undergoing dialysis now have a %MARD lower than 10%, which indicates significantly improved sensor accuracy (34). Additionally, endogenous substances like urea and uric acid, severe metabolic acidosis and extreme fluid overload are known to interfere with the glucose oxidase reaction in glucometers, may also affect sensor performance (35). MARD values also differ with different venous and capillary comparator analysers: Freestyle Libre 3 (ranges between 9.5-11.6%), Dexcom G7 (9.9-12%) and Medtronic (11.6-16.6%) (36, 37). Dexcom G6 and Freestyle Libre 2 also show excellent correlations between improvements in TIR and Hba1c (10). The UK Diabetes Specialist Nurse Forum has also published detailed technical and accuracy data in their website to facilitate updated comparisons of currently available CGM devices (38).

Amongst patients with advanced kidney impairment on PD, CGM correctly identified 96.5% of hyperglycaemic and 60% of hypoglycaemic events, with alarm detection rates of 94.9% and 100%, respectively. Further, authors found no significant correlations between MARD and BMI, extracellular water, relative hydration index, lean or fat mass, or haemoglobin levels (39). Additionally, de Boer et al. reported that CGM frequently identified hypoglycaemia and hyperglycaemia that may not be clinically evident in dialysis patients (40). A recent study evaluated the accuracy of the Dexcom G6 continuous glucose monitoring (CGM) system in hospitalised patients with diabetes (n=31) undergoing maintenance HD (41). The MARD was approximately 20% overall, with higher values observed during HD sessions (22.0%) compared to non-HD periods (18.2%). In a prospective study, Genua et al. assessed the accuracy of the FreeStyle Libre (FSL) Generation 1 intermittently scanned glucose monitoring system in patients with diabetes mellitus (n=16) undergoing maintenance haemodialysis and reported that the global MARD was 23%, increasing to 29% during HD sessions (42). Avari et al. evaluated the accuracy of two CGM systems, Dexcom G6 and Abbott Freestyle Libre 1, in 40 adults with diabetes undergoing haemodialysis (43). The overall MARD was 22.7% for Dexcom G6 and 11.3% for Freestyle Libre 1. Further, authors recently investigated the accuracy of the latest generation Dexcom G7 CGM system in adults with diabetes undergoing HD (44). Compared to laboratory measurements, the overall MARD for Dexcom G7 was 10.4% (44). Zhang et al, in a meta-analysis, acknowledges that CGM provides valuable insights into glycaemic variability and control, but robust evidence from large, well-designed randomized controlled trials remains limited and interpretation of CGM-derived data in CKD patients should be approached with caution due to potential alterations in glucose metabolism and device accuracy in this population (13).

## Kidney transplant donors

Transplant physicians often prevaricate about the ethics of kidney donation in high-risk individuals with DM, prediabetes, family history of DM and gestational DM. The lack of clarity in guidelines have perpetuated concerns about ethics of such practice, given that donors will already have a small risk of kidney disease progression with a singleton kidney. The limitations of current measures to predict future DM or DM-related complications have compounded the concerns about donor selection. International renal guidelines do not have consensus views, and permission to donate are usually left to the discretion of clinicians and local ethics committees. Position statements from international societies are often imprecise and vague. The Organ Procurement and Transplantation Network (OPTN) recently amended policy to allow kidney donations from living donors with DM in 2022 (45). Other leading guidelines made nebulous references to donation from diabetic donors: “only under exceptional circumstances’ (European Best Practice Guidelines) (46), “after a thorough assessment of the lifetime risk.” (British Transplantation Society) (47), “individualization to allow for the consideration of very low-risk individuals with Type 2 DM (T2DM)’ (KDIGO) (48). Their positions on prediabetes are also contentious: “impaired glucose tolerance is not an absolute contraindication to donation” (European Best Practice Guidelines) (46), “individualized based on demographic and health profile in relation to transplant program’s acceptable risk threshold” (KDIGO) (48). The current OPTN guideline enlists eligibility criteria for donation from preexisting type 2 DM patients, where the only objective measure of glycaemic control is “Hba1c < 7% on at least three occasions within the past 2 years” Additionally, for high-risk individuals, including prediabetes, history of gestational diabetes and first-degree relatives of diabetics, GTT or Hba1c is the only required test to justify donation eligibility (45).

Given the potential consequence of developing DM and kidney failure with solitary kidney, there should arguably be more rigorous measures to screen donors. In a 10-year follow up study, Chandran et al. showed that risk of prediabetics developing DM was higher in kidney donors compared to healthy controls (15.6% vs 2.2%) (49). Independent to DM risks, prediabetic patients are also prone to glomerular hyperfiltration (50) and development of microalbuminuria (51), although overt risk in developing CKD have not been elucidated. However, for context, a previous study involving 8280 donors with 1826 with impaired fasting glucose at the time of donation, there was no difference in patient survival and kidney failure rates in the two groups of patients (52).

The use of CGM as a screening and predictive tool for development of future DM could be valuable in the donor selection process. CGM can display GV through graphical display and project progression from prediabetes to advanced diabetes, through analysis of CV and TIR (53). Important salient features that can also be detected by CGM, which can predict onset of T2DM in prediabetic patients, are postprandial hyperglycaemia and overnight rise in blood glucose (dawn phenomenon) (54). When used in tandem with demographic factors, CGM metrics can also detect disease progression and classifying risk of Type 1 DM (T1DM), potentially defining eligibility for prevention trials (55). Machine learning technology can also enhance detection of T1DM in high-risk individuals through self-administered CGM home testing (56). Similarly, predictive potential for T2DM can be harnessed through CGM-generated glucose curves to predict muscle-insulin-resistance and β-cell-deficiency sub-phenotypes and stratify individuals with glucose dysregulation (57). Research have also highlighted that CGM can enhance self-monitoring behaviour and increase exercise adherence in prediabetics (58), which would support a preventative role for CGM in such donors.

## Preoperative, Intraoperative and intensive care usage of CGM

CGM experience within the preoperative, intraoperative and critical care phase of KT is limited. However, evidence from non-CGM studies have indicated benefits of intensified glycaemic control on outcome measures in both pre- and intra-operative phase. Amongst 2872 diabetic patients who underwent KT, poor pretransplant glycaemic control (through HbA1c measurements) was associated with decreased patient survival (59). Another study with 832 patients admitted for solid organ transplantation (561 KT and 12 KT with other organs), elevated admission glucose level amongst non-DM patients was associated with increased 30-days mortality (60). Amongst 680 liver transplantation patients, intraoperative hyperglycaemia was independently associated with postoperative infection and mortality (61).

Amongst studies utilizing CGM in KT, Hagerf et al. reported the use of CGM in 65 critically ill patients after abdominal surgery (13 of which had concomitant kidney transplant surgery with pancreas, islet cells and liver transplant) through an infraclavicular sensor placement. The study showed that CGM was safe, feasible and reliable, with promising potential to improve glycaemic management (62). Another study assessing usage of CGM in prediabetes patients during elective abdominal surgery showed acceptable and consistent accuracy during the operation with MARD of 12.7% compared to reference capillary glucose readings. Amongst recent KT patients, in a randomized controlled trial, who were intensively treated with intravenous insulin regimen versus standard subcutaneous insulin regimen, the former group had less delayed graft function (18% vs 24%), although the results were not significant (63). A prospective randomized controlled trial to evaluate the efficacy of intraoperative CGM in predicting adverse outcomes in liver transplantation is currently being conducted (64), but a previous study on liver transplantation have reported that incidence of post-operative graft dysfunction was higher in recipients with intraoperative CV of > 28% than in recipients in low CV (10.8% vs 0%, p < 0.05) (65). In cardiac surgery, Rangasami et al. demonstrated that GV through measurements of CV and blood glucose risk index values have negative prognostic outcomes for major adverse events (66).

A review of 22 studies reported high technical reliability within the intraoperative setting but highlighted concerns about accuracy (67). Perez-Gulman et al. reported electrocautery interferences, common patterns of signal loss and negative bias during surgery; but within intensive care setting, CGM use was helpful to guide insulin delivery when regular point of care tests were impractical especially in situations when hourly POC tests were required for decision-making in the sickest patients that required continuous insulin infusion (68). Mannitol, used intraoperatively as osmotic diuretic and transurethral irrigation fluids, was identified as possible interferent for the Eversense CGM sensor to falsely high readings (69).

## Postoperative usage of CGM

Hyperglycaemia occurs in about 80-90% of patients in the first days to week following KT (70). Many non-CGM studies have acknowledged that this pattern is associated with increased rates of acute rejections, high infection rates and mortality (71). Early post-transplant hyperglycaemia (within the first 45 days), is an independent risk factor for hospital readmissions, worst graft function, acute rejection and future PTDM development (72, 73). Whilst immediate poor glycaemic control was associated with poor graft outcomes, strict glycaemic control (HbA1c < 7.7%) may conversely also be detrimental (74). The need to regulate glycaemic control over a tight therapeutic range lends towards the application of rigorous CGM metrics to complement existing measures for postoperative surveillance.

Studies utilizing CGM metrics have already demonstrated interesting patterns beyond traditional HbA1c-centric studies. In a randomized controlled trial, Jandowitz et al. in 2023 showed that patients on CGM have significantly lower rates hyperglycaemic (≥ 10mmol/l) episodes and median glucose levels without increasing the number of hypoglycaemic (≤ 4.4 mmol/l) episodes, compared to those with finger-stick glucose monitoring in the first 5 days of transplant (75). Shin et al, using CGM pre- and post-operatively in KT patients for two weeks, showed that CGM has superior predictive potential for PTDM over capillary blood glucose monitoring (76). Male patients with a higher postoperative TAR of 10mmol/l were also identified as a significant risk factor (66). Another study, specifically in non-diabetic patients, demonstrated that hyperglycaemia is a constant feature in the immediate post-operative phase, with 19% of patients developing PTDM after a mean follow up of 72 months (77).

Comparing kidney and liver transplant patients, CGM showed that KT patients have greater glycaemic excursions and GV in the immediate post-op phase (78). The mean amplitude of glycaemic excursion and mean absolute glucose levels were also higher in KT patients (79). Amongst combined organ procedures, Dadlani et al. in 2019 compared simultaneous kidney pancreas (SPK), kidney after pancreas (PAK) and pancreas only transplant, with CGM providing a viable and reliable way of monitoring glycaemic control 3–6 weeks post-transplant (79). Mittal et al. also showed that amongst 22 SPK patients, CGM correlated well with post-operative OGTT and was easier to perform and provided 24-hour data that can help decision making (80).

## Long-term usage and predictive AI modelling of CGM

Good glycaemic control has been demonstrated to achieve better long-term outcomes through Hba1c-driven studies (74). Amongst CGM studies, Aouad et al. reported the evolution of glycaemic control over 0, 3 and 6 months in 28 patients post KT demonstrating a distinct pattern of afternoon and evening hyperglycaemia (81). Pasti et al. confirmed these findings in a cohort of paediatric patients whereby those with IGT tend to have “lowest glucose” level and less hypoglycaemic episodes, whereas glucose variability tends to improve with time after KT (82). Amongst long-term transplant patients, Werzowa et al. identified that existing DM patients had significant GV compared to PTDM patients, indicating potential pathophysiological differences between the two groups (83). Jakubowska et al. reported that CGM had a positive effect of KT patients with pre-existing DM or PTDM on perception of quality of health (84). However, Kurnikowski et al. showed that there was no difference in long term control amongst patients on CGM and continuous subcutaneous insulin therapy against standard of care basal insulin therapy amongst KT patients in a study with follow up of up to two years (85).

Artificial intelligence, through the use of machine learning, has been used in KT specifically with radiological and pathological evaluation of allograft, prediction of graft survival, optimizing dose of immunosuppression and prediction of graft function (86). Elefteriadis et al. reported the predictive potential of CGM for PTDM in non-diabetic KT patients at varying time points after the operation (87). The study showed good concordance with oral glucose tolerance test (OGTT) from day 8 to day 90, yielding high sensitivity/specificity especially those achieving high % TAR threshold (87). Aouad et al. also reported that the magnitude of hyperglycaemia and variability in transplant patients 3–6 months post-transplant is also predictive towards the development of PTDM during follow up through the use of the Glycaemic Risk Assessment Diabetes Equation score (81). In PD, the use of CGM alongside multinomics approach through PD fluid effluent can influence diabetic management through individualizing diet, medication and dialysis management (88). To date, there are machine-learning driven AI algorithms that are trained to interpret complex datasets from CGM tracings to predict the development of diabetic retinopathy and nephropathy, which can potentially be used for early diagnosis of complications and prediction of outcomes (89).

## CGM in acute kidney injury and the failing transplant graft

AKI frequently occurs in KT patients from a myriad of reasons, including sepsis and acute rejection (90). This review did not identify any studies investigating the utility of CGM in patients with AKI, but many studies have indicated that intensive surveillance of glycaemic control can improve outcomes. Diabetic patients have higher infection and rejection risks, on top of inherent propensity for DKA and hypoglycaemia (91). Patients with greater variability in Hba1c (HVS) are reported to have an increased risk of AKI, with a hazard ratio of 1.4 for HVS of 0-20% against 80-100% (92).

A failing transplant graft will typically show severe dysglycaemia through the effects of acidosis, uraemia and inflammation (5). The use of immunosuppressive regimens can contribute to increased GV owing to their variable pharmacodynamic effects, particularly in the context of reduced renal reserve. Corticosteroids diminish insulin sensitivity (18), while CNI induces pancreatic β-cell toxicity (17), together promoting an erratic hyperglycaemic pattern. Conversely, prolonged steroid exposure may result in iatrogenic adrenal insufficiency, leading to unpredictable hypoglycaemic episodes (93). In addition, fluctuating CNI levels in the setting of a failing graft may further impair eGFR and alter the renal clearance of oral hypoglycaemic agents. Furthermore, true glycaemic assessment is also impaired by the discordance between CGM and Hba1c readings at advanced stages of CKD (27). Frequent infections, rejection episodes, anti-rejection rescue therapy and frequent hospital admissions necessitate the need for tighter glycaemic surveillance. The 2020 KDIGO guideline has recommended the use of GMI from CGM to evaluate glycaemic control in patients with CKD4-5, in situations where Hba1c may not be reliable (7). Stathi et al. reported a case series with the use of automated insulin delivery system with CGM in SPK patients with failing graft function to tighten glycaemic variability and reduce hypoglycaemia, in order to protect residual pancreatic and kidney graft function (94).

## Research direction

There is a paucity of published research on CGM usage in KT patients, with only 18 papers identified in the literature. (**Table 2**) (62, 75–85, 87, 94–98) More research in specific domains is needed to elucidate our understanding on this subject.

**Table 2:** Summary of studies that utilizes CGM in KT patients.

First author	Year	Origin	Journal	Study type	Number of patients / range	Type of patients / type of CGM	Objectives	Results
Dmitriev I et al (85)	2023	Russia	Diagnostics	Cross-sectional obervational	43 (2014-2021)	SPKT Type 1 DM/ Medtronic	To asses TIR and GV in T1DM with pancreatic grafts at different times after SPKT	Patients showed high TIR and low GV post SPKT
Rodriguez et al (86)	2010	USA	Diabetes Technology and Therapeutics	Case control	23 ( 8 SPK, 6 KA, 9 healthy controls)	SPKT1 Type 1 DM Medtronic Minimed	To identify differences in glycemic ex cursions and frequency of hyper- and hypoglycemia among the three study groups	CGM showed that SPK is better than KA transplant in uncovering glycaemic excursions
Vantyghem MC (87)	2012	France	The Journal of Clinical Endocrinology and Metabolism	Obervational cohort study	23 (9 patients with islets after KT)	Islet transplantations Type 1 DM		CGM detects that the four components of dysglycaemia (mean glucose, standard deviation, hyperglycaemia and hypoglycaemia) were not equally affected by degree of islet cell functions
Werzowa et al (75)	2015	Austria / Germany / Italy	Journal of Diabetes and its Complications	Observational cohort study	28 (10 transplant patients without DM, 10 patients with PTDM and 8 non transplant patients with DM	Kidney transplant Type 2 DM Medtronic	To analyze glycemic control quality and variability in renal transplanted subjects both with and without PTDM	CGM showed that DM patients showed significant GV compared to PTDM patients, indicating potential pathophysiological differences between the two groups
Elefteriadis et al (88)	2024	Germany / Italy	Transplant International	Prospective cross-sectional	41 patients with no prior history of DM	Kidney Transplant Freestyle Libre	To assess feasibility of CGM and investigate its potential for the diagnosis of PTDM and IGT	CGM confirms that Hba1c can be a good indicator for IGT in KT patients
Wojtusciszyn A et al (69)	2013	France	Diabetes Metabolism	Observational cross-sectional	43 patients with no prior history of DM	Kidney Transplant Medtronic	To assess blood glucose (BG) levels immediately following kidney transplantation in non-diabetic subjects and to explore their relationship to later graft outcomes and NODAT occurrence.	CGM confirms that hyperglycaemia is a constant characteristic immediately post transplant and could identify patients with future risk of PTDM and graft failure
Dadlani et al (71)	2019	USA	Clinical Transplant	Observational Cross-sectional	11 SPK, 9 PAK, 6 PTA	Kidney pancreas transplant Type 1 DM Dexcom G4	To assess glycemic control in the first 6 weeks after pancreas transplantation using CGM a	CGM provided a reliable source of monitoring, demonstrating excellent control in all 3 types of transplants
Jin et al (70)	2019	South Korea	Journal of Diabetes Research	Observational Cross-sectional	31 (24 KT and 7 Liver transplant)	Kidney transplant Medtronic	To investigate the glucose profiles and assess the degree of hyperglycemic excursion after kidney or liver transplantation during the early period after operation. M	CGM showed that KT patients have greater degree of glucose fluctuations and occurrence of hyperglycaemia than liver transplant patients in the post-operative phase
Hagerf et al (56)	2024	Czech Republic	Diabetes Care	Observational Cross-sectional	65 (3 liver and pancreas tx, 9 kidney and pancreas, 1 kidney and ialets)	Kidney with liver, pancreas and islets Tx Dexcom G6	To assess the feasibility and accuracy of real-time con tinuous glucose monitoring (CGM) in ICU patients after major abdominal surgery.	CGM provides an accurate and reliable source of glycaemic control data in postoperative ICU patients and a feasible alternative sensor placement site
Elefteriadis et al (79)	2024	Germany / Italy / Austria	American Journal of Transplantation	Prospective longitudinal	46	Kidney transplant Freestyle Libre	To explore the potential of CGM regarding the prediction of PTDM and IGT on day 90 after transplantation in previously nondiabetic KT recipients	CGM has the potential to facilitate everyday clinical practice as a screening test by predicting by predicting the occurrence of PTDM and IGT
Jandovitz et al (67)	2023	USA	Clinical Transplant	Prospective randomized controlled trial	40	Kidney transplant Medtronic	To examine whether the use of inpatient Continuous Glucose Monitors provides improved glycemic control over finger-stick glucose monitoring post-transplant.	Patients on CGM has significantly reduced incidence of hyperglycaemic episodes and median glucose levels in the first 5 days post-transplant without increasing the number of hypoglycaemic episodes
Jakubowska et al (76)	2024	Poland	Renal Failure	Prospective cross-sectional study	8 (7 with PTDM and 1 with existing DM)	Kidney transplant (2 -23 months post-transplant) Dexcom G6	To evaluate glycemic profiles using the Dexcom G6 CGM system for 30 d a personal smartphone in people with diabetes after kidney transplantation and to assess the impact of monthly use of the CGM system on glycemic control and quality of life in this group.	CGM is identified as a useful method to monitor glycaemic control and gives transplant patients a positive impact on the perception of health
Aouad et al (73)	2018	Australia	Transplantation	Prospective cross-sectional study	28	Kidney Transplant Preexisting DM and non-DM Medtronic	To prospectively describe patterns of glycemic control and variability over a 6-month period after kidney transplantation using continuous glucose monitoring	CGM identified that magnitude of hyperglycemia and variability are maximal in recipients with preexisting diabetes and significant in those who go on to develop NODAT.
Shin et al (68)	2024	South Korea	Scientific reports	Prospective cohort	60	KT Non-diabetics Freestyle Libre 1	To identify changes in glucose levels pre- and post-KT using CGM and investigate the risk factors associated with the incidence of PTDM. Also to compare predictive efficacy of CBGM and postoperative CGM in relation to PTDM occurrence.	Postoperative CGM provides detailed glucose dynamics and demonstrates superior predictive potential for PTDM than capillary blood glucose monitoring
Mittal et al (72)	2015	United Kingdom	Transplant International	Prospective cohort	26 (22 SPK and 4 PTA)	KT Type 1 DM Medtronic	To use CGM to assess the metabolic profile of pancreas transplant recipients in the early post-transplant period.	CGM correlated well with post-operative oral glucose tolerance test and is easier to perform and provided 24 hour data that can help decision making
Kurnikowski et al (77)	2024	Austria	Kidney medicine	Open labelled randomized	85	KT No diabetes Medtronic	To assess long-term glucose control in patients treated with continuous subcutaneous insulin therapy versus standard-of-care and subcutaneous once-daily basal insulin therapy using HbA1c levels at month 3 after kidney transplantation and during the 2-year follow-up	Patients on continuous subcutaneous insulin and CGM were not superior at reducing HbA1c levels at month 3 or PTDM prevalence at months 12 and 24 compared with the control or basal insulin group
Pasti et al (74)	2013	Hungary	Paediatric Surgery	Observational	20	KT Paediatric patients	To examine the glycemic homeostasis of 20 renal-transplanted children using routine laboratory tests and CGM system	CGM showed that data show that "lowest glucose" level and hypoglycemic episodes are significantly influenced and altered in renal-transplanted patients with IGT.
Stathi D et al (84)	2023	United Kingdom	Journal of Diabetes Investigation	Case series	6 (5 with SPK)	SPK	To share experience using diabetes technology in people with type 1 DM and SPK or pancreas-only transplant post graft failure	Use of diabetic technology improved glycaemic control and should be encouraged in patients with failing pancreatic graft

SPKT, Simultaneous pancreas kidney transplantation; KA, Kidney alone; PTA, Pancreas transplant alone; PAK, Pancreas after kidney; IGT, Impaired glucose tolerance; ICU, Intensive care unit; NODAT, New onset diabetes after transplant (old terminology for PTDM); CBGM, Continuous blood glucose monitoring.

### Determination of pattern in different KT phases

CGM-metrics should be used to evaluate clinical end-points like PTDM, acute and chronic rejections, delayed graft function, graft survival, CMV and BK infections and graft survival. Patient-reported outcomes like quality of life and psychosocial modifications will be particularly useful in failing grafts and complex patients, especially those with combined pancreas transplants.

### Determination of accuracy

Similar to research in dialysis population, more studies should be done to determine accuracy of CGM in transplant patients, especially within acute settings in operating theatres and intensive care.

### Donor and recipient assessment

Donor assessment in high-risk patients can also be improved with CGM to predict future DM. Augmentation of pre-transplantation glycaemic control in recipients and its impact on post-transplantation outcomes can be explored. Prediabetic donors and recipients may also benefit from long-term CGM usage to mitigate risks and promote behavioural changes.

### Predictive algorithm

Machine-learning technology can be used to evaluate patients’ glycaemic risk profile and tailor individualised immunosuppressive regimen. More research can be done to improve existing predictive algorithms for PTDM.

### Medical education and training

There is a need to improved current understanding and knowledge of clinicians and transplant patients on CGM-metrics for glycaemic assessment and evaluate ways to incorporate CGM education to transplant and nephrology curriculum. Barriers to adoption of CGM amongst varied healthcare professionals includes lack of training/education and limited exposure (99).

### Improving access to CGM

Equity of access to CGM can be improved through more research to produce affordable alternatives (100). Additional, prioritized access can be given to selected KT population especially during high-risk periods where there is added values in utilizing CGM-metrics to improve clinical management or when alternate means of glycaemic surveillance is deemed difficult.

## Conclusion

CGM heralds a new era in DM management and surveillance, with the potential to influence short- and long-term management in both KT donors and recipients. In pre-transplant settings, CGM can complement traditional tests to determine suitability of high-risk donors for donation. There is scope for using CGM during the intraoperative and postoperative phase of the transplant, to deliver target-driven glycaemic control and prevent transplant-related complications. Additionally, CGM can circumvent hypoglycaemia and improve glycaemic variability in patients with failing grafts. More research should be done to determine accuracy and utility of CGM, harness the potential of predictive algorithms, improve patients’ and clinician’s understanding and enable equity of access to patients.
